# Avoidability of hospital deaths and association with hospital-wide mortality ratios: retrospective case record review and regression analysis

**DOI:** 10.1136/bmj.h3239

**Published:** 2015-07-14

**Authors:** Helen Hogan, Rebecca Zipfel, Jenny Neuburger, Andrew Hutchings, Ara Darzi, Nick Black

**Affiliations:** 1 Department of Health Services Research & Policy, London School of Hygiene & Tropical Medicine, London WC1H 9SH, UK; 2 Department of Surgery & Cancer, St Mary’s Campus, Imperial College, London, UK

## Abstract

**Objectives** To determine the proportion of avoidable deaths (due to acts of omission and commission) in acute hospital trusts in England and to determine the association with the trust’s hospital-wide standardised mortality ratio assessed using the two commonly used methods - the hospital standardised mortality ratio (HSMR) and the summary hospital level mortality indicator (SHMI).

**Design** Retrospective case record review of deaths.

**Setting** 34 English acute hospital trusts (10 in 2009 and 24 in 2012/13) randomly selected from across the spectrum of HSMR.

**Main outcome measures** Avoidable death, defined as those with at least a 50% probability of avoidability in view of trained medical reviewers. Association of avoidable death proportion with the HSMR and the SHMI assessed using regression coefficients, to estimate the increase in avoidable death proportion for a one standard deviation increase in standardised mortality ratio.

**Participants** 100 randomly selected hospital deaths from each trust.

**Results** The proportion of avoidable deaths was 3.6% (95% confidence interval 3.0% to 4.3%). It was lower in 2012/13 (3.0%, 2.4% to 3.7%) than in 2009 (5.2%, 3.8% to 6.6%). This difference is subject to several factors, including reviewers’ greater awareness in 2012/13 of orders not to resuscitate, patients being perceived as sicker on admission, minor differences in review form questions, and cultural changes that might have discouraged reviewers from criticising other clinicians. There was a small but statistically non-significant association between HSMR and the proportion of avoidable deaths (regression coefficient 0.3, 95% confidence interval −0.2 to 0.7). The regression coefficient was similar for both time periods (0.1 and 0.3). This implies that a difference in HSMR of between 105 and 115 would be associated with an increase of only 0.3% (95% confidence interval −0.2% to 0.7%) in the proportion of avoidable deaths. A similar weak non-significant association was observed for SHMI (regression coefficient 0.3, 95% confidence interval −0.3 to 1.0).

**Conclusions** The small proportion of deaths judged to be avoidable means that any metric based on mortality is unlikely to reflect the quality of a hospital. The lack of association between the proportion of avoidable deaths and hospital-wide SMRs partly reflects methodological shortcomings in both metrics. Instead, reviews of individual deaths should focus on identifying ways of improving the quality of care, whereas the use of standardised mortality ratios should be restricted to assessing the quality of care for conditions with high case fatality for which good quality clinical data exist.

## Introduction

For over 20 years the overall standardised mortality ratio (SMR) for all deaths in a hospital has been advocated as an indicator of the quality (encompassing both safety and effectiveness) of a hospital.^1^ Although an association between the SMR for a specific disease (such as acute myocardial infarction, pneumonia, and severe sepsis) and measures of quality of care (such as adherence to clinical guidelines) has been shown,^2^ ^3^ ^4^ ^5^ similar studies on hospital-wide SMRs have not been reported. Despite concerns about the value of hospital-wide SMRs being raised by experts in the United Kingdom,^6^ United States,^7^ Canada,^8^ and Australia,^9^ many countries have adopted them and continue to use them.

The Keogh review^10^ used hospital-wide SMRs to select acute hospital trusts (National Health Service organisations that comprise either a single hospital or a group of local hospitals) in England for detailed consideration of their quality.^10^ This review was established in February 2013 in the wake of the second Francis report into Mid-Staffordshire NHS Foundation Trust.^11^ It aimed “to review the quality of care and treatment provided by those NHS trusts and NHS foundation trusts that are persistent outliers on mortality indicators [hospital-wide SMRs].” The 14 trusts selected had a higher than expected hospital-wide SMR for two consecutive years according to either of the two widely used metrics—the hospital standardised mortality ratio (HSMR) and the summary hospital level mortality indicator (SHMI).

In July 2013, one of the main recommendations of the review was the need for a study into the relation between “excess mortality rates” (based on hospital-wide SMRs) and “actual avoidable deaths” (based on retrospective case record review by experienced clinicians).^12^ The latter was considered to provide a more meaningful indication of the quality of clinical care, being based on clinicians’ careful and detailed review of each death rather than on a statistical probability derived from routine administrative data. Although case record review should not be considered as the ideal, given its known limited reliability,^13^ at least a moderately strong association between the two measures would provide some reassurance as to the validity of hospital-wide SMRs as a measure of mortality associated with poor quality of care.

The only published account of the relation between hospital-wide SMRs and proportions of avoidable deaths was a study of 10 acute hospital trusts in England in 2009.^14^ This found no association with the HSMR (correlation coefficient −0.01).^15^ Given the small sample size, however, the 95% confidence interval was wide (−0.64 to 0.62). Subsequently, data for SHMI were obtained,^16^ which showed that the association with that metric was also not significant (−0.24, 95% confidence interval −0.76 to 0.46).

To meet the objective of the Keogh review, we enlarged that study to increase confidence in the estimate of the proportion of avoidable deaths determined by case record review and the association between the proportion of deaths that were avoidable (due to acts of omission and commission) in a trust and its HSMR and SHMI.

## Methods

The retrospective case record review design that we used was an adaptation of studies conducted in the UK, Canada, and the Netherlands.^17^ ^18^ ^19^ ^20^

### Sampling strategy

A clinically important association between hospital-wide SMR and avoidable death proportion would be indicated by a regression coefficient of at least 0.5 (that is, an increase of 1% in avoidable death proportion for an increase in HSMR of one standard deviation, assumed to be 10). We report regression coefficients rather than correlation coefficients, for the reason explained below in the description of analyses.

We determined that, based on an avoidable death proportion of 5% (SD 2%),^6^ a sample of 34 trusts and 100 cases per trust would have 80% power to detect a regression coefficient of 1.4 (that is, a 1.4 percentage point increase in avoidable death proportion for a 10 point increase in SMR). Estimates of statistical power were based on simulated two stage sampling of 34 trusts from a population of trusts, and then 100 cases per trust.

We stratified all 141 acute trusts by HSMR, and then used sampling to ensure that the trusts were representative for teaching status, size, and location. Using the hospital administration system in each of the trusts, staff trained in the study methods randomly selected the case records of 100 patients who had died in hospital during the index year. If a case record could not be found, a substitute patient was randomly selected. Information on missing case records was recorded (age, sex, admitting specialty, reason why missing) to check for sampling bias. Through assiduous searching, only 5.3% of records were missing. We excluded obstetric, psychiatric, and paediatric patients (who accounted for <5% of all hospital deaths in England and Wales in 2012).

### Definitions

For each case, reviewers were initially asked to judge whether there had been any problem in care that had contributed to the patient’s death. We defined problems in care as patient harm resulting from acts of omission (inactions such as failure to diagnose and treat according to evidence based guidelines), acts of commission (affirmative action such as incorrect treatment or management), and harm as a result of unintended or unexpected complications of healthcare. This definition was seen as more helpful than adverse event, patient safety incident, or error because it focuses beyond single discrete incidents to take a wider view of the overall quality of care provided and its contribution to a patient’s death. The definition was also more likely to ensure that deaths related to failure to act (omissions) were recognised, particularly if these occurred over days or weeks.

For each case where a problem in care had been identified, reviewers were asked to make a judgment as to the avoidability of that death. Some problems in care can result from exemplary clinical practice (for example, where there is a known risk of a complication that could lead to death such as a patient experiencing an intracerebral bleed when a thrombolytic drug had been appropriately administered after myocardial infarction) and would not be regarded as avoidable. In other cases, patients may have experienced a problem in care but their concurrent illness was so complex or severe that even if the problem had contributed to their death, the death itself was not judged avoidable during that admission.

Among the patients in whom an avoidable event had occurred, reviewers were asked to assess the likelihood of the death being avoidable on a Likert scale (table 1). For the analyses, we defined the proportion of avoidable deaths in a trust as those where the likelihood of avoidability was judged to be more than 50% (grade 4-6 on the scale). This included deaths that were “definitely preventable,” “strong evidence it was preventable,” and “probably preventable.”

**Table 1 tbl1:** Prevalence of avoidable deaths

Grade of avoidability*	Definition of grade	2009† (n=1000)	2012/13‡ (n=2400)	Overall (n=3400)
2	Slight evidence of avoidability	28 (2.8)	87 (3.6)	115 (3.4)
3	Possibly avoidable but not very likely, less than 50-50	33 (3.3)	68 (2.8)	101 (3.0)
4	Probably avoidable, more than 50-50	29 (2.9)	45 (1.9)	74 (2.2)
5	Strong evidence of avoidability	20 (2.0)	25 (1.0)	45 (1.3)
6	Definitely avoidable	3 (0.3)	1 (0.0)	4 (0.1)
4-6	>50% likelihood	52 (5.2)	71 (3.0)	123 (3.6)

*Reviewer assessed using Likert scale.

†“Was the patient’s death due to problems in the healthcare or did problems in healthcare contribute to the death?”

‡“In your judgment, is there some evidence that the patient’s death was avoidable if the problem/s in healthcare had not occurred?”

### The review process

Several activities that have been shown to improve reliability were incorporated in the review process^17^ ^18^ ^20^ ^21^ ^22^: the use of experienced clinicians, one day reviewer training, provision of written guidance, ongoing support from the principal investigator (HH), the opportunity to raise and discuss questions with a more experienced reviewer, and the use of a structured medical review form.

We recruited 67 doctors, all of whom had extensive experience as generalists (66% currently practising, and 34% recently retired), and many were already engaged in case record reviews in their own trusts. When necessary, specialist medical advice was available either from other reviewers within the group or from elsewhere. This was most often used to obtain a surgical opinion when a reviewer came from a medical specialty.

Reviewers were allocated to trusts with which they had no previous connection. As reviews took place on site, reviewers were able to request additional materials such as laboratory reports stored on computer, if these were missing from the clinical record. All deaths considered to have an element of avoidability were discussed with an expert reviewer. This aimed to reduce the risk of false positive results and increase the reliability of the decision.

The inter-rater reliability (the level of agreement between reviewers) of the judgment of avoidable deaths (grade 4-6) based on a random sample of 486 cases drawn from all trusts and subjected to double reviews was consistent with that of previous studies (κ 0.45, 95% confidence interval 0.24 to 0.66).^13^

### Medical review form

We asked the reviewers to consider all aspects of patient care, including nurses’ and allied health professionals’ notes, drug charts, and diagnostic test results. Findings were recorded on a structured medical review form. Information collected on each patient included age, sex, admitting specialty (medical; surgical), type of admission (elective; emergency), comorbidity (number of conditions and type), and overall impairment. In all cases where a problem in care was judged to have contributed to death, reviewers reported the type of problem, its timing, and any associated causative or contributory factors before making a judgment as to whether the death was avoidable. Among patients in whom there was an element of avoidability, we also asked reviewers to estimate the length of life lost. Reviewers also rated overall quality of care on a scale from very poor to excellent, using a validated method,^23^ with free text space to provide more detail. Although reviews focused on the admissions during which death occurred, reviewers were also asked to identify problems that occurred before that admission if these seemed to have contributed to a patient’s death.

The study design was similar in both data collection periods, although some minor improvements were made in 2012/13 based on our experiences in 2009. Within the medical review form: some additional information on mental capacity (dementia, mental illness, and learning difficulties) and end of life care were included; questions related to comorbidities were simplified; and the question on avoidability was rephrased to improve clarity: “Was the patient’s death due to problems in the healthcare or did problems in healthcare contribute to the death?” (2009); “In your judgement, is there some evidence that the patient’s death was avoidable if the problem/s in healthcare had not occurred?” (2012/13).

### Analyses

We entered anonymised data onto EpiData 3.1 and analysed it using Stata (versions 12 and 13) software. Data on trust HSMR were obtained from Dr Foster and SHMI from the Health and Social Care Information Centre.

Linear regression was used to investigate the association between trusts’ hospital-wide SMRs and avoidable death proportions (aggregated for the trust) rather than correlation because the correlation is subject to downward biased estimates if there is random measurement error in one of the variables. In this study the scope for random error was far greater for avoidable death proportions (owing to sampling 100 deaths for each trust) than for hospital-wide SMRs (based on all deaths and admissions in a year). In contrast, downward bias in the estimates of a regression coefficient (regression dilution bias) arises in the presence of random error in the independent variable but will not arise from random error in the dependent variable which, in this analysis, was avoidable death proportion.^24^ The (aggregated) trust proportion was used as the dependent variable. No weights were used because the sample size was the same for all trusts.

Binomial regression models for proportions that allow for extra-binomial variation were also fitted, but presented results are confined to the simpler linear regression models as the conclusions were the same. The count of avoidable deaths was used and the sample size was included as the offset.

### Patient involvement

There was no patient involvement in this study.

## Results

### Proportion of avoidable deaths

The proportion of patients where death was judged to be avoidable (more than 50% likely; grade 4-6) was 123 (3.6%, 95% confidence interval 3.0% to 4.3%, table 1). Fewer deaths were deemed avoidable in 2012/13 (3.0%, 2.4% to 3.7%) than in 2009 (5.2%, 3.8% to 6.6%).

### Association between avoidable death proportion and hospital-wide SMRs

Figure 1 shows the proportion of avoidable deaths by HSMR for the 34 trusts. Overall, based on 34 trusts, there was little evidence of an association between HSMR and the proportion of avoidable deaths in a hospital (table 2). The regression coefficient was 0.3 (95% confidence interval −0.2 to 0.7; P=0.23). Thus, a one standard deviation in HSMR such as between 105 and 115, was associated with an increase of only 0.3% (95% confidence interval −0.2% to 0.7%) in the proportion of avoidable deaths. The regression coefficient was similar in both time periods (0.1 and 0.3).

**Figure fig1:**
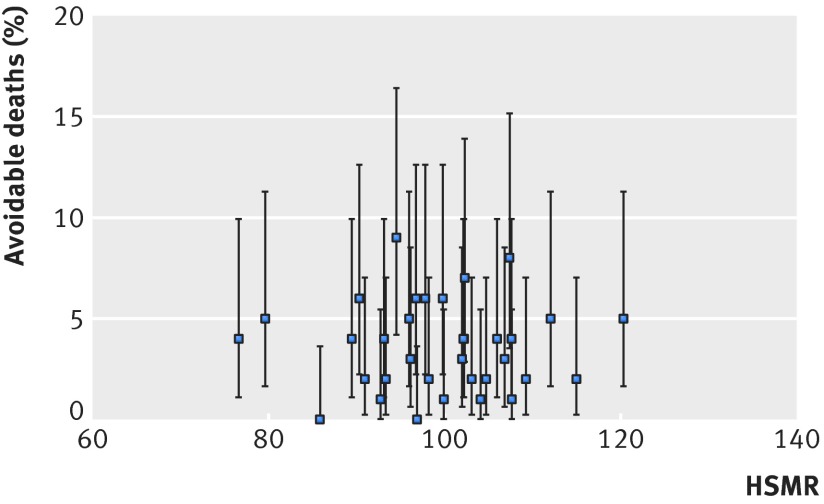
**Fig 1** Avoidable death proportion (95% confidence interval) for 34 trusts by hospital standardised mortality ratio (HSMR)

**Table 2 tbl2:** Regression coefficients (95% confidence intervals) for relation* between avoidable death proportion and two hospital-wide standardised mortality ratio (SMR) metrics, the hospital standardised mortality ratio (HSMR) and the summary hospital level mortality indicator (SHMI)

Sample of trusts	HSMR		SHMI	
	Regression coefficient (95% CI)	P value	Regression coefficient (95%CI)	P value
Overall (n=34)	0.3 (-0.2 to 0.7)	0.23	0.3 (-0.3 to 1.0)	0.29
2009 (n=10)	0.1 (-0.1 to 1.3)	0.82	-0.02 (-1.0 to 0.6)	0.56
2012/13 (n=24)	0.3 (-0.2 to 0.7)	0.26	0.5 (-0.4 to 1.3)	0.24

*Regression coefficient can be interpreted as percentage point increase in avoidable death proportion for 10 point increase in SMR.

A similar positive but non-significant association was observed for SHMI (0.3, 95% confidence interval −0.3 to 1.0). Figure 2 shows the proportion of avoidable deaths for the 34 trusts.

**Figure fig2:**
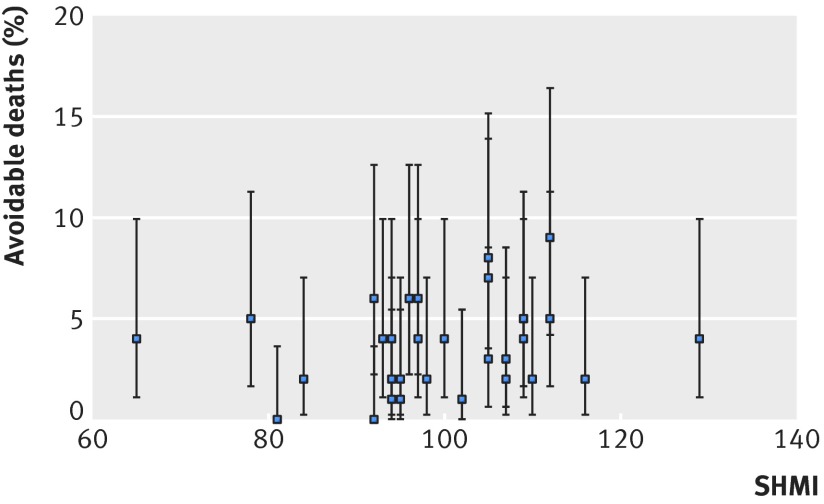
**Fig 2** Avoidable death proportion (95% confidence interval) for 34 trusts by summary hospital level mortality indicator (SHMI)

## Discussion

Only weak positive associations were observed between the proportion of avoidable deaths in a trust and two commonly used standardised mortality ratio metrics, the hospital standardised mortality ratio (HSMR) or summary hospital level mortality indicator (SHMI), neither reaching statistical significance. A difference in standardised mortality ratios (SMRs) between 105 and 115 would be associated with a difference in the proportion of avoidable deaths of only 0.3 percentage points (95% confidence interval −0.2% to 0.7%). Even if a larger sample of trusts and cases was taken, it would be unlikely to reveal a clinically important association, even if it achieved statistical significance. Thus, hospital-wide SMRs do not provide a useful indication of the proportion of avoidable deaths in a trust.

The absence of even a moderately strong association is a reflection of the small proportion of deaths (3.6%) judged likely to be avoidable and of the relatively small variation in avoidable death proportions between trusts. This confirms what others have demonstrated theoretically—that is, no matter how large the study, the signal (avoidable deaths) to noise (all deaths) ratio means that detection of significant differences between trusts is unlikely.^6^

Although there was no statistically significant difference over time in the proportion of deaths in which reviewers judged there to be an element of avoidability (that is, including even a slight possibility) between 2009 (11.3%) and 2012/13 (9.4%), there was a statistically significant difference in the proportion of deaths deemed to be more than 50% likely to have been avoidable (5.2% *v* 3.0%). An improvement in quality of care is only one of five factors that may have contributed. Firstly, in 2012/13 patients were sicker; a higher prevalence of several key comorbid conditions was reported by reviewers (for example, metastatic cancer 11.4% *v* 6.0%; chronic obstructive pulmonary disease 36.7% *v* 13.2%; heart failure 21.1% *v* 8.8%). Whether or not this was a real difference or reflected greater propensity to record these comorbidities, the impact on reviewers is likely to mean they were less likely to judge a death avoidable. Secondly, reviewers’ awareness of the use of “do not attempt resuscitation” orders was probably greater as a result of the wider use of highly visible forms in the case records plus changes to the medical review form, which drew their attention to such orders. Thirdly, there was a minor difference in the wording of the question about attribution of avoidability. And finally, a perception is that there has been a change in culture in the National Health Service over those years,^25^ which might have led to an increasing reluctance of reviewers to criticise other clinicians.

The weak positive (although non-significant) association between SMRs and avoidable death proportion seen in 2012/13, but absent in 2009, may have been that the reviewers were aware that the principal aim during this second phase of data collection was to investigate such an association. As such, they knew that the sample of trusts had been stratified according to HSMR and could easily have found out the HSMRs for the trusts they were studying. In 2009, reviewers were not made aware that the trusts were a stratified sample.

### Strengths and limitations of this study

This is the largest nationally representative retrospective case record review to have been conducted in England and one of the largest worldwide. It is the first published comparison of hospital-wide SMRs and estimates of the frequency of avoidable deaths based on detailed clinician reviews of case records.

The lack of a significant association between hospital-wide SMRs and avoidable death proportions may reflect the methodological limitations of both types of measure. Hospital-wide SMRs, being based on routine administrative data, are unable to take into account the severity of a patient’s primary condition. In addition, they are subject to variation between trusts as regards clinicians’ diagnostic practice, thoroughness of recording comorbid conditions, use of palliative or end of life coding (which affects HSMR but not SHMI), and availability of alternative facilities for patients where death is imminent. Even where adjustment for case mix is carried out in an attempt to account for some of these factors, it has the potential to increase rather than to reduce bias in the SMR as an indicator of the quality of care.^26^

Retrospective case record review using two reviewers has only moderate reliability, reflecting the subjective element in judgments of avoidability and the quality of care. Despite adopting practices known to improve reliability (training, use of a standard review form, availability of expert advice), the inter-rater reliability was only moderate (κ 0.45), similar to that reported in other studies. High reliability can only be achieved by using five or more reviewers for each case record, which was not feasible.^27^ To minimise this limitation we ensured that the 100 deaths in each trust were allocated to several reviewers from different specialist backgrounds rather than all being reviewed by one person.

Another limitation may have exaggerated the strength of any association. Although the reviewers were not informed of the trust’s HSMR or SHMI, they could easily have obtained such information. They may also have had a pre-existing view of the quality of the trust based on reputation and hearsay. Such knowledge may have influenced their judgments as to the occurrence of problems in care and the avoidability of deaths. This would have contributed to overestimating the association between hospital-wide SMR and avoidable death proportion.

Another potential limitation was restricting the reviewing to doctors. Although doctors were instructed to consider not only the medical documentation in the case records but also the documents from nurses and other professionals, the inclusion of reviewers from those backgrounds may have taken a different view of the avoidability of deaths.^28^

Finally, we chose to define avoidable as being at least a 50% likelihood of the death being avoidable, a definition that has wide credibility and acceptability. Analyses based on an even broader definition of avoidability, including those with only slight evidence of avoidability, resulted in larger although still non-significant regression coefficients with wider confidence intervals (HSMR 0.6, 95% confidence interval −0.2 to 1.5; SHMI 0.7, 95% confidence interval −0.7 to 2.0). A difference in SMRs between 100 and 115 would be associated with a difference in the proportion of such deaths of only 0.9%.

### Implications for policy

Despite the methodological limitations described, the low rate of avoidable deaths combined with the absence of a significant association between hospital-wide SMRs and avoidable death proportions suggests that neither metric is a helpful or informative indicator of the quality of a trust. It is potentially misleading to the public, clinicians, managers, and commissioners to praise or condemn a trust on the basis of either measure. In addition, although it was beyond the scope of this study, neither measure should be used as a screening test (smoke alarm) to identify poor quality trusts until its validity for that purpose has been rigorously evaluated and demonstrated.

There are, however, two ways in which consideration of hospital deaths may assist in assessing and improving quality. Firstly, there is evidence of the value of SMRs for specific groups of patients, but this requires not only that death is a frequent outcome (such as critical care or high risk major surgery) but also that high quality clinical data are available that allow adequate risk adjustment. Secondly, routinely reviewing case records of patients who die in trusts provides an opportunity for identifying local quality problems and stimulating improvements. It has clinical credibility by taking account of the complexity of patients’ conditions and care, and it can indicate whether or not poor care was responsible for any death. Although some form of mortality reviewing takes place in all acute trusts in England, standardisation of the process (selection of deaths, review forms, training of reviewers, judging avoidability) would help ensure adequate rigour throughout the hospital sector.

### Implications for research

Four potentially productive lines of inquiry could be pursued. Firstly, we will explore the relation between avoidable deaths and other measures of safety such as healthcare acquired infections, staff views of the safety of their hospital, and patient incident reports. Secondly, the validity of other possible metrics based on avoidable deaths, such as weighted means of grades of avoidability, could be explored. Thirdly, the method of determining avoidability might be improved through exploring other ways of defining and measuring the likelihood of certain events being avoidable. In addition, it would be interesting to explore the sensitivity of results to blinding reviewers to the identity of the hospital, although this would be expensive given the vast number of references to the identity of a hospital in a case record. Comparison with case records of patients who survive might also prove to be productive. Finally, by combining our data with that from other countries we could assess the impact of increasing the power of the analysis.

**What is already known on this topic**

Hospital-wide standardised mortality ratios (SMRs) are commonly used as an indicator of a hospital’s quality but have not been validated
The proportion of hospital deaths judged to be avoidable based on retrospective case record review has been reported to be about 4-5%
The association between hospital-wide SMRs and the proportion of avoidable deaths is uncertain; one study found no association but was too small to provide definitive evidence

**What this study adds**

The lack of a statistically significant association between hospital-wide SMRs and the proportion of avoidable deaths was confirmed
Both hospital-wide SMRs and avoidable death proportions based on the judgment of only one or two reviewers have methodological shortcomings making them unsuitable indicators to compare the quality of hospitals

## Notes

We thank the staff of the 34 trusts for participating and supporting the case record reviewing; the clinicians for reviewing the records; Mike Campbell for providing summary hospital level mortality indicator data for 2009; and Frances Healey, Graham Neale, Richard Thomson, Charles Vincent, and three peer reviewers for providing valuable advice and comments.

Ethical approval: This study was approved by the NRES Committee London-Central. Research governance approval was granted by each participating trust.

Data sharing: No additional data available.

Transparency: The lead author (HH) affirms that this manuscript is an honest, accurate, and transparent account of the study being reported; that no important aspects of the study have been omitted; and that any discrepancies from the study as planned have been explained.

## Notes

Cite this as: *BMJ* 2015;351:h3239
